# A generic outcome assessment of mobility capacity in neurorehabilitation: measurement properties of the de Morton Mobility Index

**DOI:** 10.1186/s12883-021-02327-0

**Published:** 2021-07-28

**Authors:** Tobias Braun, Detlef Marks, Christian Thiel, Christian Grüneberg

**Affiliations:** 1 Department of Applied Health Sciences, Division of Physiotherapy, Hochschule für Gesundheit (University of Applied Sciences), Gesundheitscampus 6-8, 44801 Bochum, Germany; 2 IB University of Health and Social Sciences, Study Center Cologne, Cologne, Germany; 3 Physiotherapy Department, Rehaklinik Zihlschlacht, Hauptstr. 2, 8588 Zihlschlacht, Switzerland; 4 Faculty of Sports Science, Training and Exercise Science, Ruhr-University Bochum, Bochum, Germany

**Keywords:** Neurological rehabilitation, Outcome assessment, Mobility limitation, Reproducibility of results, Rasch analysis, Validity

## Abstract

**Background:**

Mobility capacity is a key outcome domain in neurorehabilitation. The de Morton Mobility Index (DEMMI), an established and generic outcome assessment of mobility capacity in older patients, is promising for use in neurorehabilitation. The aim of this study was to examine the measurement properties of the DEMMI in rehabilitation inpatients with neurological conditions.

**Methods:**

Cross-sectional study including a mixed sample of adult inpatients in a neurorehabilitation hospital. Structural validity, unidimensionality and measurement invariance (Rasch analysis), construct validity, internal consistency reliability, and inter-rater reliability of the DEMMI (scale range: 0–100 points) were established. The minimal detectable change, the 95% limits of agreement, and possible floor and ceiling effects were calculated to indicate interpretability.

**Results:**

We analyzed validity (n = 348) and reliability (n = 133) in two samples. In both samples, the majority of participants had a sub-acute stroke or Parkinson’s disease.

Rasch analysis indicated unidimensionality with an overall fit to the model (chi-square = 59.4, P = 0.074). There was no relevant measurement invariance by disease group. Hypotheses-based correlation analyses (DEMMI and other functional outcome assessments) showed sufficient construct validity. Internal consistency reliability (Cronbach’s alpha = 0.94) and inter-rater reliability (intraclass correlation coefficient = 0.94; 95% confidence interval: 0.91–0.95) were sufficient. The minimal detectable change with 90% confidence was 15.0 points and the limits of agreement were 39%. No floor or ceiling effects were observed.

**Conclusions:**

Results indicate sufficient measurement properties of the DEMMI in rehabilitation inpatients with neurological conditions. The DEMMI can be used as a generic outcome assessment of mobility capacity in neurorehabilitation.

**Trial registration:**

German Clinical Trials Register (DRKS00004681). Registered May 6, 2013.

**Supplementary Information:**

The online version contains supplementary material available at 10.1186/s12883-021-02327-0.

## Background

For individuals with neurological conditions, mobility limitations are a frequent and critical issue which negatively affect independence in daily living and quality of life, and increase the risk of falls [1, 2]. Thus, improvements in mobility capacity, especially in walking and balancing, are considered the most important rehabilitation goals of patients with neurological conditions [3–5].

Although guideline-directed interventions vary for different neurological conditions, such as stroke or Parkinson’s disease (PD) [6, 7], patients are often treated in inpatient or outpatient rehabilitation facilities which are not focused on a single disorder. In such settings, generic outcome assessments are used to measure outcome domains that are common in many disease groups. For example, the Functional Independence Measure is used as generic measure of disability across different neurological conditions [8, 9].

Mobility capacity, too, is a health-related outcome domain relevant to neurorehabilitation that is often assessed across different disease groups with generic outcome assessments. However, commonly used assessments, such as timed walking tests for gait speed, the timed up and go test, and the 6-min walk test [5, 10–12], cover only a limited range of mobility components (e.g., walking on plane surface) and their clinical usability is limited, because they require the patient’s ability to walk. Thus, a considerable number of individuals cannot be assessed, particularly in the early and sub-acute stages of rehabilitation after an acute neurological event, such as a stroke [13, 14]. Ceiling effects also impact clinical utility, particularly when an assessment is intended to measure progress over the duration of recovery [10].

An ideal generic outcome assessment of mobility capacity in neurorehabilitation needs to fulfil the following characteristics: performance-based, measure on interval level, affordable, easy to learn for assessors, feasible, safe, valid over the whole mobility spectrum, sound measurement properties (e.g. validity, reliability, responsiveness), and invariant across disease groups and other patient characteristics [12]. A clinical outcome assessment that fulfils these requirements in geriatric care is the de Morton Mobility Index (DEMMI) [15]. The DEMMI was developed based on the Rasch model [16, 17] to measure the mobility capacity of older hospital patients [15] – a heterogenous population of individuals with a broad range of limitations in self-care, mobility, and cognition. This performance-based clinical outcome assessment has a broad scale width, covering low- to high-order mobility abilities and producing interval-level test scores. The DEMMI’s measurement properties have been examined in various health conditions and care settings, indicating sufficient validity, reliability, responsiveness to change and unidimensionality in acute and sub-acute older patients [15, 18–20], osteoarthritis [21], hip fracture [22] dementia [23–25], and critically ill patients [26]. Some studies provide very promising evidence that the DEMMI is a feasible, valid, reliable and unidimensional assessment for individuals with neurological conditions, such as stroke [27, 28], PD [29, 30], and mixed neurological conditions [27]. The DEMMI form fits on one paper sheet and can be administered by health professionals within 5─10 min without special equipment [15, 20, 23].

However, the DEMMIs suitability as a generic outcome assessment of mobility capacity and most of its measurement properties in neurorehabilitation have never been investigated. We hypothesized that the DEMMI is a generic outcome assessment of mobility capacity in neurorehabilitation and we aimed to evaluate the DEMMI’s measurement properties in a mixed sample of rehabilitation inpatients with neurological conditions.

## Methods

Reporting of this study was informed by the STROBE guideline for observational studies, the GRRAS guideline for reliability studies and the criteria of the COSMIN risk of bias checklist [31–34].

### Design and setting

We performed a cross-sectional study of the DEMMI’s measurement properties in neurorehabilitation. This study was approved by the Local Committee for Ethics in Medical Research (Canton of Thurgau, Switzerland: 2013/13), registered a priori (German Clinical Trials Register: DRKS00004681), performed according to the ethical principles described in the Declaration of Helsinki, and all participants gave written informed consent. All methods were performed in accordance with the relevant guidelines and regulations.

Briefly, rehabilitation inpatients with neurological conditions were examined with the DEMMI and a set of functional assessments (listed below) on several occasions during their rehabilitation course to analyze the DEMMI’s psychometric properties. The present study reports on the DEMMI’s structural and construct validity, internal consistency, inter-rater reliability, measurement error, interpretability, and feasibility for the complete sample of rehabilitation inpatients with neurological conditions. The DEMMI’s measurement properties for sub-samples of the total trial sample with stroke (n = 121) and PD (n = 116) have been published previously [28, 29].

The study was conducted in a neurological rehabilitation hospital in Switzerland, where patients were typically referred from acute hospitals, neurologist consultants, or general practitioners located in the eastern and central parts of Switzerland.

### Participants

**Fig. 1:**
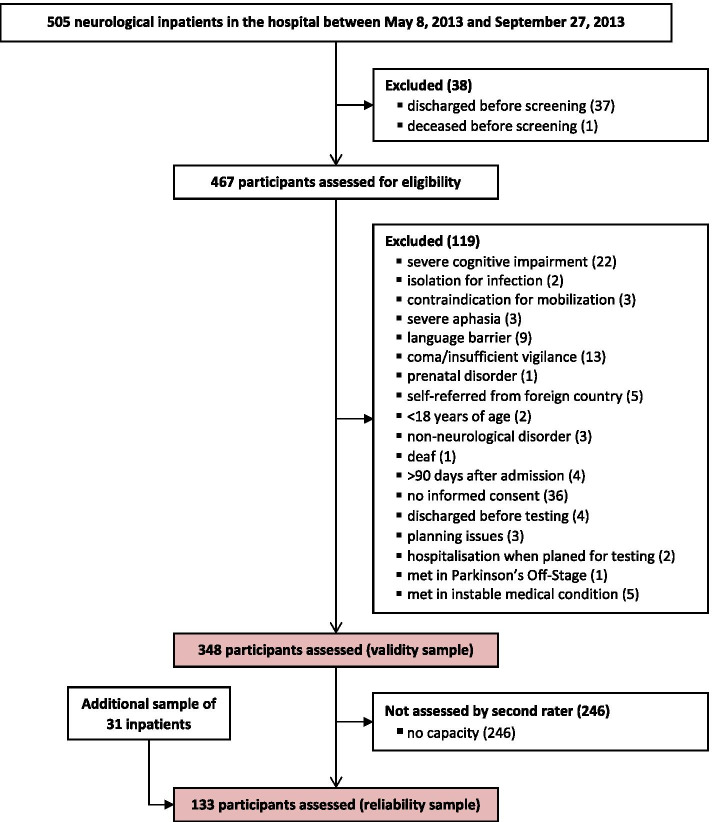
Flow chart of study participants

The study sample consisted of all inpatients present on May 8, 2013 or entering the rehabilitation hospital consecutively within the following 20 weeks. Inclusion criteria were a neurological disorder and an age of 18 years and older. The main exclusion criteria were severe cognitive impairment and a contraindication for mobilization (for all criteria, see Fig. 1).

### Procedures

Eligible participants were examined by the primary investigator (TB) in a single session of 30─45 min scheduled within the first 7 days after hospital admission, if possible. The DEMMI and a comprehensive set of functional assessments were performed in a standardized order (baseline).

The participants’ socio-demographic data were taken from the medical records. For common disorders, disease-specific measures were performed to describe disease severity and functional capacity. For participants with stroke, the National Institutes of Health Stroke Scale was assessed to measure the global severity of stroke symptoms [35]. For participants with PD and Multiple Sclerosis, Hoehn and Yahr staging [36] and the Expanded Disability Status Scale [37] were completed by the hospital neurologist, respectively. In all three scales, higher scores indicate higher impairment or disease severity.

Inter-rater reliability was examined between 2 trained and experienced physiotherapists, the primary investigator (TB) and a second rater (DM). Characteristics of both raters are described elsewhere [28, 29].

The second rater performed the DEMMI independently in a convenient sub-sample (reliability sample). Participant selection was mainly based on the second rater’s availability (temporal resources) and on participants’ consent to perform a second study assessment. Both DEMMI assessments were performed within 2 days. To create a stable retest situation, participants were excluded if they reported a change in their physical or mental condition with respect to the first session (e.g., fatigue, pain, ON/OFF state in PD). The test environment (patient’s room) was similar for both sessions (baseline and retest). Both raters were blinded toward each other’s ratings and we tried to balance the number of participants each rater visited first.

A sample size of ≥ 50 participants for reliability studies has been proposed to be “good” at the times of study conduction [38, 39]. However, within the initial recruitment period (20 weeks), we could not include ≥ 50 participants for each major sub-sample of participants with stroke and PD, respectively. Hence, we set up a second recruitment period, using the same inclusion criteria, and screened all present and incoming patients over a period of 9 consecutive days. This additional sample of convenience was only included in the inter-rater reliability analysis.

### Measurements

Participants were assessed with the DEMMI, together with a set of functional assessments, including Berg Balance Scale, timed up and go test, 10-m walk test, Functional Ambulation Categories (FAC), 6-min walk test, Performance Oriented Mobility Assessment, and Functional Independence Measure. For the sub-samples of participants with stroke and PD, we performed additional functional assessments, which were only used to analyze these sub-samples [28, 29].

A detailed description of the assessment procedures and a description of the comparator assessments are given in the Additional file 1. Table 1 provides an overview of the scale width and constructs measured by the comparator assessments.

**Table 1:** Construct validity of the de Morton Mobility Index (n = 348) including the hypotheses on construct validity and the constructs of the comparison measurement instruments

No	Hypothesis	Comparison measurement instrument			Observed correlation with DEMMI (Spearman’s correlation)		Hypothesis confirmed
		Measurement instrument	Construct	Mean ± SD (range) or median (IQR)	rho	95% CI	
1	A correlation of > 0.7 was expected between the DEMMI and other measures of mobility and functional independence	POMA, 0–28 points	Mobility	18 ± 10 (0–28)	0.94	0.93 to 0.95	Yes
2		Timed Up and Go test (n = 266), sec^a^	Mobility	14 ± 11 (4–76)	0.80	0.75 to 0.84	Yes
3		10-m gait speed, fast (n = 277), m/sec	Mobility	1.15 ± 0.51 (0.11–2.00)	0.75	0.69 to 0.80	Yes
4		FIM mobility sub-scale (n = 325), 5–35 points	Mobility	23 ± 8 (5–35)	0.80	0.76 to 0.84	Yes
5		FAC, 0–5 points	Ambulation	4 (3–5)	0.89	0.87 to 0.91	Yes
6		6-min walk test (n = 276), meters	Walking endurance	349 ± 161 (28–664)	0.82	0.78 to 0.86	Yes
7		Berg Balance Scale, 0–56 points	Balance	36 ± 19 (0–56)	0.95	0.94 to 0.96	Yes
8		FIM total (n = 325), 18–126 points	Functional independence	86 ± 24 (18–126)	0.73	0.68 to 0.78	Yes

*SD* standard deviation, *IQR* interquartile-range, *CI* confidence interval, *DEMMI* de Morton Mobility Index, *POMA* Performance Oriented Mobility Assessment, *FIM* Functional Independence Measure, *FAC* Functional Ambulation Categories

^a^ indicates hypothesis of a negative correlation

### DEMMI

The DEMMI is a performance-based clinical outcome assessment of mobility capacity, consisting of 15 hierarchical mobility items [15, 20, 40, 41]. The patient is asked to perform functional tasks related to bed and chair mobility, ambulation, static balance, and dynamic balance. The items are rated with 2-or 3-point response options, resulting in a maximum ordinal score of 19 points. This raw score is transformed into a total interval DEMMI score of 0–100 points, with higher scores indicating a higher level of mobility capacity.

### Statistical analysis

Data were analyzed using SPSS version 23.0 and Microsoft Excel (Professional Plus 2016) for all analyses except the Rasch analysis, which was completed using RUMM2030 version 5.1 software. Descriptive statistics were used to present sample characteristics. Interval-based data were examined for normal distribution with the Shapiro–Wilk test of normality and by visual inspection of the related histograms and P-P-plots. The DEMMI scores were not normally distributed (p < 0.001); therefore, only non-parametric statistics were applied. A significance value of 5% was used.

## Measurement properties

### Structural validity (Rasch analysis)

The Rasch model is a probabilistic model asserting that item response is a logistic function of item difficulty and person ability [16]. The DEMMI was developed based on the Rasch model in geriatric inpatients [15] and data fitted the model in various other medical conditions [20, 22, 23, 28, 30].

We performed a Rasch analysis to evaluate the following properties of the DEMMI in neurological inpatients: stochastic (probabilistic) ordering of items, monotonicity (increase in item responses consistent with the underlying trait), local item independence (zero correlation between items when conditioned on the score), unidimensionality, and group invariance (no difference in response to item by group membership when at the same level of (in this case) ‘mobility capacity’), which is also called differential item functioning (DIF). Data fit to the model was deemed acceptable if a set of criteria was fulfilled (Additional file 1). Full details of the Rasch analysis process are given elsewhere [17, 42]. Reporting followed established recommendations [17].

A target sample size of at least 150 was set to provide 99% confidence within ± 0.5 logits [43]. The unrestricted (partial credit) Rasch polytomous model was used with a conditional pair-wise parameter estimation.

### Construct validity

In absence of a ‘gold standard’ for ‘mobility capacity’, construct validity was assessed by following the methodological approach of hypotheses testing [38, 39]. We used the other functional outcomes and participants’ clinical information to assess the DEMMI’s construct validity. Aspects of convergent and known-groups validity were used to formulate 11 hypotheses (H1–H15) [39, 44]. All hypotheses were formulated a priori, based on existing literature, and the clinical expertise of clinicians and the research team [15, 20, 23, 30, 45]. Formulated and shortened versions of the hypotheses are presented in Additional file 1 and Table 1, respectively. Details on the statistical analyses and interpretation of hypotheses testing are given in Additional file 1. A sample size of ≥ 100 participants is recommended [46].

### Reliability

Cronbach’s alpha and the Person-Item-Separation Index, which are measures of internal consistency reliability in case of a unidimensional scale, were derived from the validity sample because of its larger size [39]. An outcome between 0.70 and 0.95 was considered acceptable [39].

Inter-rater reliability was examined using the intra-class correlation coefficient (ICC) model 2.1 (two-way random effects model; ICC_AGREEMENT_) [44]. An ICC of ≥ 0.7 or higher was deemed acceptable [39]. The standard error of measurement (SEM_AGREEMENT_) was calculated and deemed satisfactory if it was ≤ 10% of the total scale range (100 DEMMI points) [44, 47]. The absolute and relative agreement between both raters per DEMMI item was calculated as a percentage (%) and as the weighted kappa with linear weights (ƙ) [44]. Agreement per item equal or above 70% and ƙ ≥ 0.70 was considered acceptable [39]. For additional information on reliability statistics, see Additional file 1.

### Interpretability

Bland and Altman’s method was used to illustrate agreement between the two raters [48]. The minimal detectable change (MDC) with 90% and 95% confidence was calculated for individual subjects (MDC_ind_) as well as for comparisons of mean scores between groups (MDC_group_) [44, 49]. A floor or ceiling effect was considered if ≥ 15% of the participants scored the highest or lowest possible DEMMI score [39]. Additional file 1 gives more information on the statistical methods.

### Feasibility

We calculated the mean administration time for the DEMMI in minutes and related the administration time to the participants’ functional status. We documented any adverse events, such as falls, reports of pain, atypical and severe changes of muscle tone, or significant fatigue.

## Results

Of 505 neurological inpatients screened for eligibility, 348 (69%) were assessed within the first recruitment period for the validity sample. For the inter-rater reliability analysis (n = 133), 102 participants could be reassessed and an additional sample of 31 participants was recruited. Figure 1 shows the flow of participants throughout the study. Table 2 gives the participants’ demographics according to psychometric sampling.

**Table 2:** Baseline characteristics of the participants by sample

Characteristic		Validity sample (n = 348)	Reliability sample (n = 133)
Age in years		66 ± 13 (18–90)	66 ± 14 (18–85)
Male/female		218/130 (63/37%)	75/58 (56/44%)
Disease-specific measures			
	Sub-acute stroke: NIH-SS, 0–42 points	5 ± 5 (0–22); n = 109	5 ± 5 (0–22); n = 51
	Parkinson’s disease: Hoehn and Yahr scale, 0–5 stages	3 (3–4); n = 100	3 (3–4); n = 47
	Multiple Sclerosis: EDSS, 0–10 grades	6.5 (6–8); n = 18	6.5 (5–8); n = 8
Disease duration,^a^ days			
	mean, SD (range)	30 ± 23 (7–150); n = 173	31 ± 23 (7–118); n = 63
	median (IQR)	22 (15–38); n = 173	22 (16–37); n = 63
Disease duration,^a^ years			
	mean, SD (range)	11 ± 8 (1–44); n = 175	12 ± 19 (1–44); n = 70
	median (IQR)	9 (5–14); n = 175	10 (5–16); n = 70
Time since admission at baseline assessment in days			
	mean, SD (range)	7 ± 12 (0–87)	11 ± 15 (0–84)
	median (IQR)	4 (2–7)	6 (3–12)
Walking aid			
	None	162 (47%)	59 (44%)
	Rollator/walker	83 (24%)	37 (28%)
	One cane/stick	25 (7%)	12 (9%)
	Two crutches/walking sticks	14 (4%)	5 (4%)
	Not ambulatory/wheelchair	64 (18%)	20 (15%)
de Morton Mobility Index, points		58 ± 26 (0–100)	59 ± 25 (0–100)

*NIH-SS* National Institutes of Health Stroke Scale, *EDSS* Expanded Disability Status Scale, *SD* standard deviation; IQR: interquartile range

Values are mean ± SD (range), median (IQR) or absolute numbers (%)

^a^ Disease duration is reported in days for (sub-acute) conditions with an exact date of event, such as stroke or spinal cord injury. Disease duration is reported in years for chronic conditions such as Parkinson’s disease or Multiple Sclerosis

The participants’ mean age was 66 ± 13 years, 218 (63%) were male, and 230 (66%) were able to ambulate independently in the hospital (FAC level ≥ 4). Most participants had a stroke (nontraumatic intracerebral hemorrhage or cerebral infarction; n = 126; 36%), including 109 (31%) in the sub-acute phase (< 6 months after stroke onset) and 17 (5%) in the chronic phase (≥ 6 months). Of 108 (31%) participants with an extrapyramidal or movement disorder, 100 (29%) had PD. Other frequent disorders were multiple sclerosis (n = 18; 5%), neoplasms of the brain or the central nervous system (n = 18; 5%) and traumatic brain injury (n = 13; 4%). Fifteen (4%) participants presented with a non-traumatic spinal cord injury due to various diseases, such as intervertebral disc disorders. The detailed sample composition according to ICD-10 diagnoses is presented in the table in Additional file 2.

In 78% of the participants, the study assessment was performed within the first 7 days after admission. There were no missing items for any DEMMI assessment. The distribution of DEMMI scores is illustrated in the figure in Additional file 2. Table 1 includes the mobility-related outcomes for all comparator assessments.

### Structural validity (Rasch analysis)

Rasch analysis was performed on the complete DEMMI item sets of 348 participants and on the complete 15-item scale. Summary fit statistics are given in Table 3. There was overall fit to the model, including no mis-fitting persons and no mis-fitting items. We found no disordered thresholds, indicating that the responses to the items were consistent with the metric estimate of the underlying construct of mobility capacity. Unidimensionality was confirmed and data were free of local dependency. Overall, the participants exhibited a higher level of mobility (mean: 2.1 logits) than the scale average (0.0 logits; person-item distribution map in Additional file 2).

**Table 3:** Summary Fit Statistics for Rasch analyses

	Item residual		Person residual		Chi-Square fit statistic		Reliability		Unidimensionality
	Mean	SD	Mean	SD	Value and (df)	P	PSI	Alpha	t-test; % (95% CI) < 5%
Results	-0.47	0.47	-0.08	0.01	59.4 (45)	0.074	0.90	0.94	2.2% (0.2 – 4.7)
Ideal values	0	1.0	0	1.0		0.003^a^	> 0.7	> 0.7	< 5.0%

^a^: Bonferonni adjustment alpha level for the DEMMI scale with 15 items (0.05/15); *SD* standard deviation, *df* degrees of freedom, *PSI* Person-item separation index, *CI* confidence interval

There was no DIF (measurement invariance) by sex, age, or disease phase (sub-acute and chronic). There was uniform DIF by disease group (stroke, PD, and ‘other’) for two items. Participants with PD were less likely to achieve item #2 ‘roll’ (found it harder; F = 7.8, p < 0.001) and more likely to achieve item #15 ‘jump’ (found it easier; F = 9.7, p < 0.001) than participants in the other two diagnosis groups, respectively (figures of the Item Characteristic Curves in Additional file 2). Further assessment indicated that both items showed ‘real’ (and no artificial) DIF [50].

The importance of DIF exhibited by item #2 and #15 was further assessed, as recommended [51], by comparing the Rasch estimate between a ‘pure’ dataset (excluding these 2 items) and a fully anchored dataset. There were trivial, non-significant differences in mean individual person logits between the two datasets (without DIF items mean: 2.34 ± 4.55, with DIF items mean: 2.26 ± 3.94, p = 0.076). In total, 33.0% of the person estimates differed by more than 0.5 logits, but the correlation between the two sets of person estimates was quite high (ICC = 0.981, 95% CI: 0.976–0.984). Overall, the results indicate that the identified DIF was unimportant.

The DEMMI’s item hierarchy in the sample of inpatients in neurorehabilitation compared to that of the development sample of geriatric inpatients [15] is illustrated in the figure in Additional file 2. A high positive logit location (e.g., tandem standing with eyes closed) indicates harder item difficulty compared to a negative logit location (e.g., sit unsupported). Deviations from the original item hierarchy are indicated by non-overlapping 95% confidence bands in 6 items.

### Construct validity

All 11 (100%) a priori stated hypotheses about correlations of the DEMMI with other clinical outcome assessments and known-group differences were confirmed. Correlations between the DEMMI and other broad outcome measures of mobility, ambulation, walking endurance, balance and functional independence (H1–H8; Table 1) were between 0.73 and 0.94. Groups of participants who walked without a walking aid (H9), who were independent in walking (H10), and who were able to climb stairs (H11) had significantly higher DEMMI mean scores than the less able comparison groups (Additional file 2).

### Reliability

Cronbach’s alpha and the Person-item Separation Index of the DEMMI were 0.94 and 0.90, respectively, indicating excellent internal consistency reliability.

For the inter-rater reliability analysis, the DEMMI assessment was performed twice by two different physiotherapists on 133 participants. Sample characteristics are given in Table 2. Rater 1 (TB) administered the first DEMMI measure in 85 (64%) participants. The two DEMMI assessments were performed on the same day in 77 (58%) participants, and within 2 days in 56 (42%) participants. There was no statistically significant mean difference in DEMMI scores between both assessors (0.1 points; 95% CI: -1.5–1.6; P = 0.92) and there was no considerable variance (0.3) due to systematic differences between the two raters. The variance between participants was 616.7 and the residual variance was 42.3. The ICC_AGREEMENT_ was 0.94 (95% CI: 0.91–0.95).

### Measurement error

The SEM_AGREEMENT_ was 6.5 points and considered acceptable (6.5% of the total DEMMI scale range).

The absolute and relative agreement per item are presented in the table in Additional file 2. There was no DEMMI item with absolute agreement < 70% (range: 80% to 99%), but 3 items with ƙ < 0.7 (range: 0.29 to 0.92).

### Interpretability

**Fig. 2:**
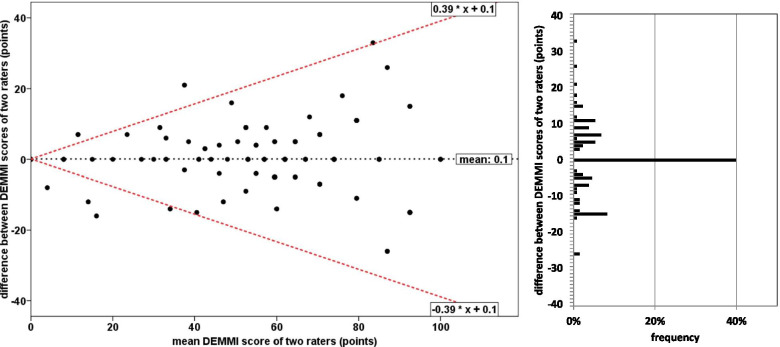
Bland–Altman plot of de Morton Mobility Index (DEMMI) scores by two raters. The x-axis represents the mean sores of the raters and the y-axis represents the difference between the raters. The dotted black line represents the mean difference between both measures; dotted red lines represent the 95% upper and lower limits of agreement. The bar chart on the right side illustrates the frequency of differences between the two raters

The Bland–Altman plot is illustrated in Fig. 2. The data were heteroscedastic (τ = 0.26) and differences were not normally distributed (P < 0.01). The 95% limits of agreement were 0.39X + 0.1 and -0.39X + 0.1, respectively, with X denoting the mean score.

The MDC_ind90_, MDC_ind95_, MDC_group90_, and MDC_group95_ were 15.0, 18.0, 1.3, and 1.6 points, respectively.

There were no absolute floor or ceiling effects, with 15 (4%) participants scoring 0 and 21 (6%) participant scoring 100 DEMMI points, respectively (histogram in Additional file 2).

### Feasibility

The mean administration time of 100 DEMMI assessments was 6.3 ± 2.1 (range: 1–14) minutes (figure in Additional file 2). DEMMI administration took ≤ 10 min in 96% (n = 333) of participants. In non-ambulant or dependent walkers (FAC ≤ 3, n = 118) and independent walkers (FAC ≥ 4, n = 230), the administration time was 6.8 ± 2.7 and 6.0 ± 1.6 min, respectively. No adverse events occurred in any DEMMI assessment.

## Discussion

This study provides evidence of the DEMMI’s sound structural and construct validity, internal consistency, inter-rater reliability, measurement error, interpretability, and feasibility in a mixed sample of rehabilitation inpatients with neurological conditions.

Rasch analysis confirmed structural validity in terms of unidimensionality, hierarchical order, measurement invariance, and logistic item structure. This is in keeping with results of other studies supporting the DEMMI’s structural validity examined in geriatric [15, 20, 22, 23] and neurological populations [28–30]. Two facets seem notably important because of their clinical relevance. Unidimensionality indicates that the DEMMI measures one single underlying construct (mobility capacity). Measurement invariance (no DIF) indicates that DEMMI items do not function differently for different members of a sample group (e.g., for men and women or for different disease groups). No (important) DIF was observed by sex, age, disease phase or disease group; therefore, clinicians and researchers can be confident that the DEMMI is a ‘fair’ test. That means, that every patient/person with the same level of mobility capacity has the same change to endorse each DEMMI items and exceed the same final DEMMI score.

The DEMMI’s construct validity in neurorehabilitation is indicated by strong correlations with other commonly applied and validated outcome assessments of mobility, ambulation, walking endurance, balance, and functional independence. The following results strengthen the conclusion of sufficient construct validity: All hypotheses were confirmed; even the lower confidence bounds of most correlations were > 0.7; and the difference in DEMMI mean scores between clinical groups was larger than the minimal important change of 10 DEMMI points [15]. This result is not surprising, since strong correlations with other measures of mobility have been reported consistently in older patients and individuals in neurorehabilitation [15, 27–29].

Cronbach’s alpha (0.94) was within the proposed acceptable range of 0.70 to 0.95 and can be judged as excellent [39]. The inter-rater reliability of the DEMMI performed by two experienced physiotherapists was 0.94 (95% CI: 0.91–0.95) and is comparable to other inter-rater reliability estimations between 0.85 and 0.94 reported by others [15, 18, 21, 23]. An ICC of ≥ 0.7 is considered sufficient for group comparisons, and a value of ≥ 0.90 is an indicator of acceptable reliability for individual-level monitoring [39, 52].

Although the ICC is quite high, the DEMMI is not free of measurement error. The SEM (6.5 points) is considered acceptable and comparable to other estimations (4.1 to 7.5 points) [20, 28, 29]. In addition, there was no item with absolute agreement < 70%. This evidence of sufficient inter-rater reliability has some crucial clinical implications in those clinical situations, in which the DEMMI is assessed on a single patient twice by two different assessors (e.g., different physiotherapists on admission and discharge). In this situation, provided that both assessors carefully synchronize before clinical use (agree on standardized administration procedures), one can be very confident that each DEMMI value represents the ‘true’ level of mobility capacity of that individual patient at that time, and that the different assessors would obtain similar scores.

We used the reliability data to establish information on the DEMMI’s interpretability [38]. We found relatively large limits of agreement (39%), which were in line with previous values reported for patients with PD (31%) [29], sub-acute stroke (42%) [28], and sub-acute geriatric conditions (-8.4 to 11.8 points) [20]. The MDC_ind90_ value of 15.0 points is considerably higher than the MDC_ind_ range of 6 to 10 points reported for older adults [15, 18–20, 24], but comparable to the MDC_ind_ values of 12.5 and 17.5 reported for the sub-samples with stroke and PD, respectively [28, 29]. Thus, a DEMMI change score, assessed by two different assessors at two different time points, needs to be ≥ 15 points (or ± 39%) to have high confidence that this change score is free of measurement error.

A possible explanation for this relatively large MDC value (and limits of agreement) could be the high variability of mobility capacity in the reliability sample (standard deviation of 25 points = 42% of the sample’s mean score). A further explanation could be that the calculation of MDC values performed here include the inter-rater variance and the participants’ intra-individual variance. Thus, the comparably large MDC values might be biased and overestimated by the included inter-rater variance. Our MDC estimations should be considered with caution and verified by future studies, which should use test–retest reliability estimations for stable patients generated by a single assessor [44, 49].

This study provides evidence for the DEMMI’s high feasibility over the whole mobility spectrum of individuals with neurological conditions, since no floor or ceiling effects occurred at hospital admission. Other authors also reported no floor or ceiling effects on admission in samples of patients with PD and stroke [27–30]. This might be an important advantage of the DEMMI over other established outcome assessments of mobility and ambulation in neurorehabilitation. We observed significant floor effects (approximately 20% of participants not able to perform these assessments) for the Timed Up and Go test, gait speed assessment, and the 6-min walk test (Table 1). With these assessments, longitudinal monitoring of mobility capacity from admission to a later point in rehabilitation would not have been possible. Similar floor effects of gait assessments in patients with stroke have been reported [13, 14]. However, a mild ceiling effect (19%) for the DEMMI in patients with stroke at hospital discharge has been reported in one study [27]. In the present sample, only 6% of participants scored the highest DEMMI score of 100 points at hospital admission. The DEMMI includes some high-level mobility items (e.g., jump, tandem standing with eyes closed) and further research is needed to evaluate whether patients who complete these items successfully (and reach the maximum score) suffer from subjective or objective mobility limitations at all; especially compared to healthy individuals of the same age.

The mean administration time of 6.3 min achieved by an experienced assessor is comparable to previous findings [15, 20, 23, 28, 29]. Based on existing evidence and our clinical experience, the DEMMI can be completed within 5–10 min in most neurological inpatients by a trained healthcare professional. High feasibility, information on interpretability, and short administration times of outcome assessments facilitate routine clinical application and enlarge therapy time [53].

### Strengths and limitations

We examined a broad set of measurement properties in a sufficiently large [43, 46] and consecutive sample of hospital inpatients with neurological conditions, supporting the generalizability of results. The included participants presented with a wide spectrum of disability, age range (18–90 years), disease duration, sub-acute and chronic conditions, and various diseases. However, the external validity of this study might be limited because the data were collected in a single rehabilitation hospital only and, with respect to prevalence estimations for central Europe, participants with PD were over-represented, whereas other conditions, such as Multiple Sclerosis and spinal cord injury, were underrepresented [54–56].

We used a combination of modern methods of latent trait theory (Rasch analysis) and methods of classical test theory. Rasch analysis provides an especially powerful tool to analyze unidimensionality, measurement invariance, and logistic item structure [17].

We did not evaluate the DEMMI’s floor and ceiling effects at any later time of rehabilitation (e.g., hospital discharge). As previously mentioned, MDC values and limits of agreement need to be interpreted with caution and might be lower than reported in this study for test–retest situations.

### Implications for clinical practice and further research

This study provides evidence that the DEMMI has sufficient key measurement properties in neurorehabilitation, including structural validity and internal consistency reliability. It seems feasible and safe, since no adverse events occurred during or immediately after test administration, which took only 6 min on average. The lack of any floor or ceiling effects on hospital admission indicates clinical value and applicability across the whole mobility spectrum of inpatients with neurological conditions. For the DEMMI administration, no long training period is required, no special equipment is needed, and there is no license charge. These advantages address some barriers to the use of outcome assessments by healthcare professionals [53, 57], and they could facilitate the application of this instrument in clinical care.

We found no relevant DIF by disease phase and disease group, indicating that the DEMMI measures were free of measurement invariance in this sample and that the DEMMI can be used as a generic measure of mobility capacity in this population.

Further research should focus on measurement properties that are still unknown in neurorehabilitation, such as test–retest reliability, responsiveness, minimal important change values, and prognostic validity. Replication of our findings is recommended [58] and should also evaluate the DEMMI’s psychometric properties in mixed samples with other compositions, including more individuals of other disease groups.

Since there are many assessments available to measure mobility capacity in neurorehabilitation, the DEMMI’s psychometric quality and clinical utility should be compared to other (generic) assessments in clinical trials and systematic reviews that follow recommended methods [59]. Future studies should investigate the DEMMI’s clinical utility for goal setting and guiding rehabilitation strategies.

## Conclusions

The DEMMI seems to be a unidimensional, valid, and reliable performance-based clinical outcome assessment of mobility capacity in adult individuals with neurological conditions that can be used generically in this population. Provided that the high feasibility, clinical utility, and sufficient measurement properties found in this study are confirmed in future studies, the DEMMI might become the standard assessment of mobility capacity in neurorehabilitation.

## Supplementary Information

**Additional file 1**. Additional information on study methods.

**Additional file 2**. Additional results.

## Data Availability

The datasets used and analysed during the current study are available from the corresponding author upon reasonable request.

## Abbreviations

DEMMI: De Morton Mobility Index
DIF: Differential item functioning
FAC: Functional Ambulation Categories
ICC: Intraclass correlation coefficient
MDC: Minimal detectable change
PD: Parkinson’s disease
SEM: Standard error of measurement
