# Supplementary material for: A generic outcome assessment of mobility capacity in neurorehabilitation: measurement properties of the de Morton Mobility Index
Source: BMC Neurol. 2021 Jul 28;21:298. doi: 10.1186/s12883-021-02327-0 (PMC8317343; doi:10.1186/s12883-021-02327-0)
Supplement: Supplementary file 1 — Additional file 1. Additional information on study methods. [file 12883_2021_2327_MOESM1_ESM.pdf]

Additional file 1: Additional information on study methods

A generic outcome assessment of mobility capacity in neurorehabilitation: measurement properties of the de Morton Mobility Index

Tobias Braun[1,2]*, Detlef Marks[3], Christian Thiel[1,4], Christian Grüneberg[1]

[1]Hochschule für Gesundheit Bochum (University of Applied Sciences), Department of Applied Health Sciences, Division of Physiotherapy, Bochum, Germany

[2]IB University of Health and Social Sciences, Study Center Cologne, Cologne, Germany

[3]Rehaklinik Zihlschlacht, Physiotherapy Department, Zihlschlacht, Switzerland

[4]Ruhr-University Bochum, Faculty of Sports Science, Training and Exercise Science, Bochum, Germany

***Correspondence:**

Tobias Braun, Hochschule für Gesundheit (University of Applied Sciences), Department of Applied Health Sciences, Division of Physiotherapy, Gesundheitscampus 6-8, 44801 Bochum, Germany. Email: tobias.braun@hs-gesundheit.de. Phone: +49-234-77727629. Fax: +49-234-77727829

Additional file 1: Additional information on study methods

Detailed description of the assessment procedures and comparator instruments

All outcome assessments were performed in the participant's hospital room and on the ward. All outcome assessments were administered by the principal investigator (TB), except noted otherwise. Instructions were given verbally. If necessary, and if this was allowed according to the formal assessment instructions, individual items or tasks were demonstrated by the assessor. Participants used the usual walking aid prescribed by the responsible physiotherapist. The same device was used for all assessments in a single session.

Similar items in different assessments were only performed once to reduce participant's burden, e.g., standing with both feet together is required in the de Morton Mobility Index (DEMMI), Performance Oriented Mobility Assessment (POMA), and the Berg Balance Scale (BBS). Breaks were offered between assessments. A hand-held digital stopwatch was used for all temporal outcomes. Walking distances were recorded with a digital measuring wheel. For participants requiring some kind of physical assistance during ambulation (Functional Ambulation Categories (FAC) scored ≤ 2), the walking tests were scored as "unable" (Timed Up and Go test (TUG), gait speed, 6-minute walk test (6minWT)) or "0" (POMA gait sub-scale), respectively. Stand-by assistance was provided for all participants whenever needed.

Most applied assessments (BBS, TUG, gait speed, 6minWT) are deemed to be sufficiently valid for measuring mobility and physical functioning in neurorehabilitation since they are recommended in clinical guidelines [1, 2]. Evidence for the validity of the other assessments is described in the following.

Berg Balance Scale (BBS)

The BBS [3] is a psychometrically sound assessment of balance for use in neurorehabilitation [4, 5]. The patient's performance in 14 static and dynamic balance tasks is rated on an ordinal scale, with lower points indicating poorer balance.

Timed Up and Go test (TUG)

The TUG assesses basic mobility functions as it asks the patient to stand up from a chair, walk 3 m, turn around, walk back, and return to the chair [6, 7]. A familiarization trial was performed, followed by two counted trials, of which the mean (in sec) was used as the final TUG score. A shorter TUG time indicates higher mobility.

10-meter walk test

Fast gait speed (m/sec) was assessed over a distance of 10 m. Participants started 90 cm prior to the starting line and were instructed to continue walking 2 meters behind the finishing line. They were timed from the moment their first foot crossed the starting line until their first foot crossed the finish line.

Additional file 1: Additional information on study methods

6-minute walk test (6minWT)

The 6minWT captures the distance in meters walked over a period of 6 minutes on a plain walkway [8]. Longer distances indicate a better mobility and walking endurance. Breaks were offered if needed.

Functional Ambulation Categories (FAC)

The clinician-completed FAC distinguishes 6 levels of walking ability subjected to the amount of assistance required over a walking distance of 10 m [9]. Lower scores, where physical assistance is needed, indicate poorer mobility than higher scores, where the patient is able to ambulate independently. Adequate predictive validity as well as excellent concurrent validity and reliability have been reported for patients with stroke [10].

Performance Oriented Mobility Assessment (POMA)

Tinetti's POMA is a clinician-rated measure of mobility and fall risk, consisting of two sub-scales [11]. Scores are summed for the balance and the gait sub-scale. A total POMA score of 28 points indicates higher mobility. There is evidence for the POMA's (moderate) construct validity in individuals with PD and stroke [12, 13].

Functional Independence Measure (FIM)

On an ordinal scale, composed of 18 items, a patient's independence in several activities is each rated on a 7-point Likert scale ranging from 1 (total dependence) to 7 (total independence) [14]. Higher scores indicate better functioning. In this study, the total score as well as the mobility subscale were used. The latter included the following 5 items: bed to chair transfer, toilet transfer, shower transfer, locomotion, stairs. Therefore, the highest achievable FIM mobility subscale score was 35 points. The FIM was administered as part of usual care by the nursing staff. Acceptable to high construct validity and unidimensionality of the FIM in neurorehabilitation has been reported [15, 16].

Additional file 1: Additional information on study methods

Additional information on the statistical analyses

Criteria for the Rasch analysis

The unrestricted (partial credit) Rasch polytomous model was used with a conditional pair-wise parameter estimation and two class intervals. Overall fit of data to the model is deemed acceptable if the following criteria are fulfilled (adopted from Mills et al. [17]:

(1) Both total chi-square probability and individual item chi-square probability values non-significant.
(2) Individual item fit residual, by convention, within ± 2.5.
(3) Mean and standard deviation of both item fit residual and person fit residual approaching 0 and 1, respectively.
(4) Ordered item category thresholds.
(5) Person-item separation index (PSI) (reliability) greater than 0.70 for group use and 0.85 for individual use.
(6) Unidimensionality (all items reflecting a single underlying latent trait) by independent t-test at the person abilities showing less than 5% of tests to be significant or the lower bound of a binomial 95% confidence interval of the observed proportion overlaps 5% [18].
(7) Pearson correlation coefficients between item residuals between 0.2 and 0.3 above the average of all item residual correlations (local independence) [19]. If the correlation was between 0.2 and 0.3 above the average, a subtest analysis using the correlated items was undertaken. Local dependence was considered if the internal consistency (PSI) of the whole item set differed substantially from the PSI in the subtest.
(8) Differential Item Functioning (DIF) occurs when different groups within the sample (e.g., women and men) respond in a different manner to an individual item, despite equal levels of the underlying characteristic (ability) being measured. ANOVA probability for differential item functioning (DIF) non-significant (5% alpha with Bonferroni correction) for the following factors: sex (male and female), age (split at median: 18 to 72 years and 73+ years), age (split at quartiles: 18 to 58 years, 59 to 68 years, 69 to 75 years and 76+ years), disease phase (sub-acute (< 6 months) and chronic (> 6 months)) and diagnosis (stroke, Parkinson's disease and 'other'). This is undertaken with a two-way ANOVA with class interval (grouped level of mobility) and the external factor (e.g. sex) as main effects. Uniform DIF is then for the main effect of the factor (e.g. gender; and there is another for class interval) and non-uniform DIF is the interaction between class interval and the factor. For DIF analysis, clinically meaningful groups of approximately equal sizes are needed.

Additional file 1: Additional information on study methods

The importance of any identified DIF was tested using a method outlined by Tenant and Pallant [20], as described by Twiss et al. [21]: "If DIF is identified it is necessary to assess the extent to which it influences the calculation of the Rasch estimates. If DIF is minor, its influence on estimates may be only slight. Rasch estimates are first calculated using a 'pure' dataset where items exhibiting DIF are removed. These estimates are then saved to an anchor file. The whole dataset, including items exhibiting DIF, are anchored to this dataset so that the estimates are defined by the measurement framework of the 'pure' items. The resulting estimates (pure vs. full anchored dataset) are then compared. The proportion of estimates that differ by 0.5 logits is calculated to assess for the proportion of non-trivial DIF" [21]. We calculated the distribution of the estimates (including mean, standard deviation), correlation between estimates (intraclass correlation coefficient), and differences between the estimates (t-test).

Construct validity

Formulated hypotheses:

- H1 – H4: DEMMI scores correlate strongly (> 0.7) with other measures of mobility capacity (Performance Oriented Mobility Assessment, Timed Up and Go test, fast gait speed, mobility subscale of the FIM), as reported for people with Parkinson's disease (PD) [22, 23], people with stroke [24], and older people [25–28].
- H5: DEMMI scores correlate strongly (> 0.7) with the Functional Ambulation Categories (FAC), a measure of ambulation. Others reported Spearman's rho correlations between DEMMI and FAC of 0.92 [28] and 0.87 [29] in older people, 0.93 in people with stroke [24] and 0.80 in people with Parkinson's disease [23].
- H6: DEMMI scores correlate strongly (> 0.7) with the 6-minute walk test, a measure of walking endurance. Others reported Spearman's rho correlations between DEMMI and 2- or 6-minute walk test of 0.76 [30], 0.70 [28] and 0.76 [29] in older hospital patients. In people with stroke, the correlation was 0.91 [24] and in inpatients with Parkinson's disease, it was 0.76 [23].
- H7: DEMMI scores correlate strongly (> 0.7) with the Berg Balance Scale, a measure of balance. In people with PD and stroke, correlations from 0.84 to 0.96 have been reported [22–24].
- H8: DEMMI scores correlate strongly (> 0.7) with the FIM total score, a measure of functional independence and disability in the activities of daily living (ADL). De Morton et al. [25] and Braun et al. [23, 24] reported correlations between the DEMMI and ADL disability of 0.68 (Barthel Index; older people), 0.85 (FIM, people with stroke) and 0.63 (FIM, people with Parkinson's disease), respectively.
- H9: Ambulatory participants (FAC ≥ 3) ambulating without a walking aid have statistically significant higher DEMMI scores than participants using a walking aid.

Additional file 1: Additional information on study methods

- H10: Independent walkers (FAC ≥ 4) have statistically significant higher DEMMI scores than non-ambulatory participants or dependent walkers (FAC ≤ 3).
- H11: Participants who can climb stairs independently (FIM stair item 6 – 7 points) have statistically significant higher DEMMI scores than participants who are dependent or cannot climb stairs (FIM stair item 1 – 5 points).

We applied one-tailed Spearman's rho analyses because directions of the correlations were hypothesized *a priori*. A one-sided Mann Whitney U test for independent samples was used to compare groups as hypotheses were formulated *a priori*. For each hypothesis on known-groups validity, we expected a statistically significant difference between the DEMMI mean scores of both groups.

Between the DEMMI and the TUG, in which lower scores represent better functioning, a negative correlation was hypothesized. The expected strengths of correlations are always reported unidirectional to improve readability.

We decided against defining an a priori threshold of e.g. ≥ 75% of hypotheses which need to be confirmed in order for a measurement instrument to be valid [31, 32]. Along with others [33], we do not think that the broad concept of construct validity can be judged as "good" or "bad" according to an arbitrary threshold of confirmed hypotheses of varying importance. Instead, we leave it to the reader to decide which percentage of confirmed hypotheses is deemed acceptable.

Inter-rater reliability

The $ICC_{AGREEMENT}$ was calculated by dividing the systematic differences between the ''true'' scores of participants by the error variance consisting of the systematic differences between the ''true'' scores of participants, the variance due to systematic differences between raters and the residual variance [34].

Measurement error

The standard error of measurement ($SEM_{AGREEMENT}$) was calculated using the same variance components used for the $ICC_{AGREEMENT}$ calculation and by taking the square root of the variance between the raters and the error variance of the $ICC_{AGREEMENT}$ [34]. The SEM was satisfactory if it was ≤ 10% of the total scale range (100 DEMMI points) [35].

Interpretability: Limits of agreement/Bland and Altman plot

The method of Bland and Altman was used to illustrate agreement between two raters [36]. The 95% limits of agreement require homoscedasticity and normally distributed differences [37]. A positive Kendall's tau (τ) correlation between the absolute differences and the corresponding means > 0.1 was

deemed to denote heteroscedasticity [38]. In case of heteroscedastic data, the following formula was used to calculate the limits of agreement: $-2X\,\frac{(10^{a}-1)}{(10^{a}+1)}\;and+2X\,\frac{(10^{a}-1)}{(10^{a}+1)}$, with a = 95% limits of agreement of the 10log transformed data and X the mean score [39].

Interpretability: Minimal Detectable Change

The minimal detectable change (MDC_{ind}) with 90% and 95% confidence, a quantification of absolute agreement, was calculated based on the inter-rater reliability data as $MDC_{ind90}=1.64*\sqrt{2}*SEM_{AGREEMENT}$ and $MDC_{ind95}=1.96*\sqrt{2}*SEM_{AGREEMENT}$, respectively. The MDC_{ind95} (MDC_{ind90}, respectively) is defined as the minimal amount of change that needs to occur between repeated assessments in an individual to exceed, with 95% (90%) confidence, the error of the measurement [40]. The MDCgroup was calculated by dividing the MDCind by the square root of the number of subjects in the sample ($\frac{MDC_{ind}}{\sqrt{n}}$) [34].

Additional file 1: Additional information on study methods

References

1. Keus S, Munneke M, Graziano M, Paltamaa J, Pelosin E, Domingos J, et al. European physiotherapy guideline for Parkinson's disease. The Netherlands: KNGF/ParkinsonNet; 2014.
2. Royal Dutch Society for Physical Therapy. KNGF Clinical Practice Guideline for Physical Therapy in patients with stroke. 12th ed. Amersfoort, The Netherlands: KNGF; 2014.
3. Berg KO, Wood-Dauphinee SL, Williams JI, Maki B. Measuring balance in the elderly: validation of an instrument. Can J Public Health. 1992;83 Suppl 2:11.
4. Qutubuddin AA, Pegg PO, Cifu DX, Brown R, McNamee S, Carne W. Validating the Berg Balance Scale for patients with Parkinson's disease: a key to rehabilitation evaluation. Arch Phys Med Rehabil. 2005;86:789–92. doi:10.1016/j.apmr.2004.11.005.
5. Blum L, Korner-Bitensky N. Usefulness of the Berg Balance Scale in stroke rehabilitation: a systematic review. Phys Ther. 2008;88:559–66. doi:10.2522/ptj.20070205.
6. Podsiadlo D, Richardson S. The timed "Up & Go": a test of basic functional mobility for frail elderly persons. J Am Geriatr Soc. 1991;39:142–8.
7. Hafsteinsdottir TB, Rensink M, Schuurmans M. Clinimetric properties of the Timed Up and Go Test for patients with stroke: a systematic review. Top Stroke Rehabil. 2014;21:197–210. doi:10.1310/tsr2103-197.
8. Enright PL, McBurnie MA, Bittner V, Tracy RP, McNamara R, Arnold A, Newman AB. The 6-min walk test: a quick measure of functional status in elderly adults. Chest. 2003;123:387–98.
9. Holden MK, Gill KM, Magliozzi MR, Nathan J, Piehl-Baker L. Clinical gait assessment in the neurologically impaired. Reliability and meaningfulness. Phys Ther. 1984;64:35–40.
10. Mehrholz J, Wagner K, Rutte K, Meissner D, Pohl M. Predictive validity and responsiveness of the functional ambulation category in hemiparetic patients after stroke. Arch Phys Med Rehabil. 2007;88:1314–9. doi:10.1016/j.apmr.2007.06.764.
11. Tinetti ME. Performance-oriented assessment of mobility problems in elderly patients. J Am Geriatr Soc. 1986;34:119–26.
12. Kegelmeyer DA, Kloos AD, Thomas KM, Kostyk SK. Reliability and validity of the Tinetti Mobility Test for individuals with Parkinson disease. Phys Ther. 2007;87:1369–78. doi:10.2522/ptj.20070007.
13. Canbek J, Fulk G, Nof L, Echternach J. Test-retest reliability and construct validity of the tinetti performance-oriented mobility assessment in people with stroke. J Neurol Phys Ther. 2013;37:14–9. doi:10.1097/NPT.0b013e318283ffcc.
14. Keith RA, Granger CV, Hamilton BB, Sherwin FS. The functional independence measure: a new tool for rehabilitation. Adv Clin Rehabil. 1987;1:6–18.
15. Ward I, Pivko S, Brooks G, Parkin K. Validity of the stroke rehabilitation assessment of movement scale in acute rehabilitation: a comparison with the functional independence measure and stroke impact scale-16. PM R. 2011;3:1013–21. doi:10.1016/j.pmrj.2011.08.537.
16. Maritz R, Tennant A, Fellinghauer C, Stucki G, Prodinger B. The Functional Independence Measure 18-item version can be reported as a unidimensional interval-scaled metric: Internal construct validity revisited. J Rehabil Med. 2019;51:193–200. doi:10.2340/16501977-2525.
17. Mills RJ, Pallant JF, Koufali M, Sharma A, Day S, Tennant A, Young CA. Validation of the Neurological Fatigue Index for stroke (NFI-Stroke). Health Qual Life Outcomes. 2012;10:51. doi:10.1186/1477-7525-10-51.

18. Tennant A, Pallant JF. Unidimensionality Matters! (A Tale of Two Smiths?). Rasch Measurement Transactions. 2006;20:1048–51.
19. Christensen KB, Makransky G, Horton M. Critical Values for Yen's Q3: Identification of Local Dependence in the Rasch Model Using Residual Correlations. Applied Psychological Measurement. 2017;41:178–94. doi:10.1177/0146621616677520.
20. Tennant A, Pallant JF. DIF matters: A practical approach to test if differential item functioning makes a difference. Rasch Measurement Transactions. 2007;20:1082–4.
21. Twiss J, McKenna SP, Graham J, Swetz K, Sloan J, Gomberg-Maitland M. Applying Rasch analysis to evaluate measurement equivalence of different administration formats of the Activity Limitation scale of the Cambridge Pulmonary Hypertension Outcome Review (CAMPHOR). Health Qual Life Outcomes. 2016;14:57. doi:10.1186/s12955-016-0462-2.
22. Johnston M, de Morton N, Harding K, Taylor N. Measuring mobility in patients living in the community with Parkinson disease. NeuroRehabilitation. 2013;32:957–66. doi:10.3233/NRE-130919.
23. Braun T, Marks D, Thiel C, Menig A, Grüneberg C. An investigation of the measurement properties of the de Morton Mobility Index for measuring mobility capacity in hospital patients with Parkinson's disease. Clin Rehabil. 2021;35:423 –435. doi:10.1177/0269215520966472.
24. Braun T, Marks D, Thiel C, Grüneberg C. Reliability and validity of the de Morton Mobility Index in individuals with sub-acute stroke. Disabil Rehabil. 2019;41:1561-1570. doi:10.1080/09638288.2018.1430176.
25. de Morton NA, Davidson M, Keating JL. The de Morton Mobility Index (DEMMI): an essential health index for an ageing world. Health Qual Life Outcomes. 2008;6:63. doi:10.1186/1477-7525-6-63.
26. Jans MP, Slootweg VC, Boot CR, de Morton NA, van der Sluis G, van Meeteren NL. Reproducibility and validity of the Dutch translation of the de Morton Mobility Index (DEMMI) used by physiotherapists in older patients with knee or hip osteoarthritis. Arch Phys Med Rehabil. 2011;92:1892–9. doi:10.1016/j.apmr.2011.05.011.
27. Braun T, Schulz R-J, Reinke J, van Meeteren NL, de Morton NA, Davidson M, et al. Reliability and validity of the German translation of the de Morton Mobility Index (DEMMI) performed by physiotherapists in patients admitted to a sub-acute inpatient geriatric rehabilitation hospital. BMC Geriatr. 2015;15:1660. doi:10.1186/s12877-015-0035-y.
28. Braun T, Grüneberg C, Thiel C, Schulz R-J. Measuring mobility in older hospital patients with cognitive impairment using the de Morton Mobility Index. BMC Geriatr. 2018;18:100. doi:10.1186/s12877-018-0780-9.
29. Braun T, Grüneberg C, Coppers A, Tofaute L, Thiel C. Comparison of the de Morton Mobility Index and Hierarchical Assessment of Balance and Mobility in older acute medical patients. J Rehabil Med. 2018;50:292-301. doi:10.2340/16501977-2320.
30. de Morton NA, Harding KE, Taylor NF, Harrison G. Validity of the de Morton Mobility Index (DEMMI) for measuring the mobility of patients with hip fracture during rehabilitation. Disabil Rehabil. 2013;35:105–11. doi:10.3109/09638288.2012.705220.
31. Terwee CB, Bot SDM, de Boer MR, van der Windt DAWM, Knol DL, Dekker J, et al. Quality criteria were proposed for measurement properties of health status questionnaires. J Clin Epidemiol. 2007;60:34–42. doi:10.1016/j.jclinepi.2006.03.012.

Additional file 1: Additional information on study methods

32. Prinsen CAC, Mokkink LB, Bouter LM, Alonso J, Patrick DL, Vet HCW de, Terwee CB. COSMIN guideline for systematic reviews of patient-reported outcome measures. Qual Life Res. 2018;27:1147–57. doi:10.1007/s11136-018-1798-3.
33. Reeve BB, Wyrwich KW, Wu AW, Velikova G, Terwee CB, Snyder CF, et al. ISOQOL recommends minimum standards for patient-reported outcome measures used in patient-centered outcomes and comparative effectiveness research. Qual Life Res. 2013;22:1889–905. doi:10.1007/s11136-012-0344-y.
34. de Vet HCW, Terwee CB, Mokkink LB, Knol DL. Measurement in medicine: A practical guide. Cambridge, New York: Cambridge University Press; 2011.
35. van Bloemendaal M, Bout W, Bus SA, Nollet F, Geurts AC, Beelen A. Validity and reproducibility of the Functional Gait Assessment in persons after stroke. Clinical Rehabilitation. 2019;33:94-103. doi:10.1177/0269215518791000.
36. Bland JM, Altman DG. Statistical methods for assessing agreement between two methods of clinical measurement. Lancet. 1986;1:307–10.
37. Altman DG, Bland JM. Measurement in Medicine: The Analysis of Method Comparison Studies. The Statistician. 1983;32:307–17. doi:10.2307/2987937.
38. Brehm MA, Scholtes VA, Dallmeijer AJ, Twisk JW, Harlaar J. The importance of addressing heteroscedasticity in the reliability analysis of ratio-scaled variables: An example based on walking energy-cost measurements. Dev Med Child Neurol. 2012;54:267–73. doi:10.1111/j.1469-8749.2011.04164.x.
39. Euser AM, Dekker FW, Le Cessie S. A practical approach to Bland-Altman plots and variation coefficients for log transformed variables. Journal of Clinical Epidemiology. 2008;61:978–82. doi:10.1016/j.jclinepi.2007.11.003.
40. Stratford PW, Binkley JM, Riddle DL. Health status measures: strategies and analytic methods for assessing change scores. Phys Ther. 1996;76:1109–23.
