# Supplementary material for: A generic outcome assessment of mobility capacity in neurorehabilitation: measurement properties of the de Morton Mobility Index
Source: BMC Neurol. 2021 Jul 28;21:298. doi: 10.1186/s12883-021-02327-0 (PMC8317343; doi:10.1186/s12883-021-02327-0)

Additional file 2: Additional results

A generic outcome assessment of mobility capacity in neurorehabilitation: measurement properties of the de Morton Mobility Index

Tobias Braun[1,2]*, Detlef Marks[3], Christian Thiel[1,4], Christian Grüneberg[1]

[1]Hochschule für Gesundheit Bochum (University of Applied Sciences), Department of Applied Health Sciences, Division of Physiotherapy, Bochum, Germany

[2]IB University of Health and Social Sciences, Study Center Cologne, Cologne, Germany

[3]Rehaklinik Zihlschlacht, Physiotherapy Department, Zihlschlacht, Switzerland

[4]Ruhr-University Bochum, Faculty of Sports Science, Training and Exercise Science, Bochum, Germany

***Correspondence:**

Tobias Braun, Hochschule für Gesundheit (University of Applied Sciences), Department of Applied Health Sciences, Division of Physiotherapy, Gesundheitscampus 6-8, 44801 Bochum, Germany. Email: tobias.braun@hs-gesundheit.de. Phone: +49-234-77727629. Fax: +49-234-77727829

Additional file 2: Additional results

Diagnoses of participants according to ICD-10 version 2014 classification

Chapter	Code	Description	Validity sample (n = 348)		Reliability sample (n = 133)	
			n	percent	n	percent
I Certain infectious and parasitic diseases	A30-A49	Other bacterial diseases	4	1.1	0	0
	A80-A89	Viral infections of the central nervous system	8	2.3	1	0.8
	B00-B09	Viral infections characterized by skin and mucous membrane lesions	3	0.9	1	0.8
	B50-B64	Protozoal diseases	1	0.3	0	0
II Neoplasms	C00-C97	Malignant neoplasms	18	5.2	3	2.3
III Diseases of the blood and blood-forming organs and certain disorders involving the immune mechanism	D80-D89	Certain disorders involving the immune mechanism	1	0.3	0	0
IV Endocrine, nutritional and metabolic diseases	E70-E90	Metabolic disorders	1	0.3	0	0
VI Diseases of the nervous system	G00-G09	Inflammatory diseases of the central nervous system	2	0.6	0	0
	G10-G14	Systemic atrophies primarily affecting the central nervous system	2	0.6	0	0
	G20-G26	Extrapyramidal and movement disorders	108	31.0	50	37.6
	G30-G32	Other degenerative diseases of the nervous system	6	1.7	2	1.5
	G35-G37	Demyelinating diseases of the central nervous system	18	5.2	8	6.0
	G40-G47	Episodic and paroxysmal disorders	2	0.6	1	0.8
	G50-G59	Nerve, nerve root and plexus disorders	0	0	0	0
	G60-G64	Polyneuropathies and other disorders of the peripheral nervous system	10	2.9	2	1.5
	G70-G73	Diseases of myoneural junction and muscle	1	0.3	0	0
	G80-G83	Cerebral palsy and other paralytic syndromes	2	0.6	1	0.8
	G90-G99	Other disorders of the nervous system	8	2.3	2	1.5
IX Diseases of the circulatory system	I60-I69	Cerebrovascular diseases	127	36.5	58	43.6
XIII Diseases of the musculoskeletal system and connective tissue	M40-M54	Dorsopathies	9	2.6	1	0.8
XIX Injury, poisoning and certain other consequences of external causes	S00-S09	Injuries to the head	17	4.9	3	2.3

Additional file 2: Additional results

Distribution of DEMMI scores for the validity and the reliability sample

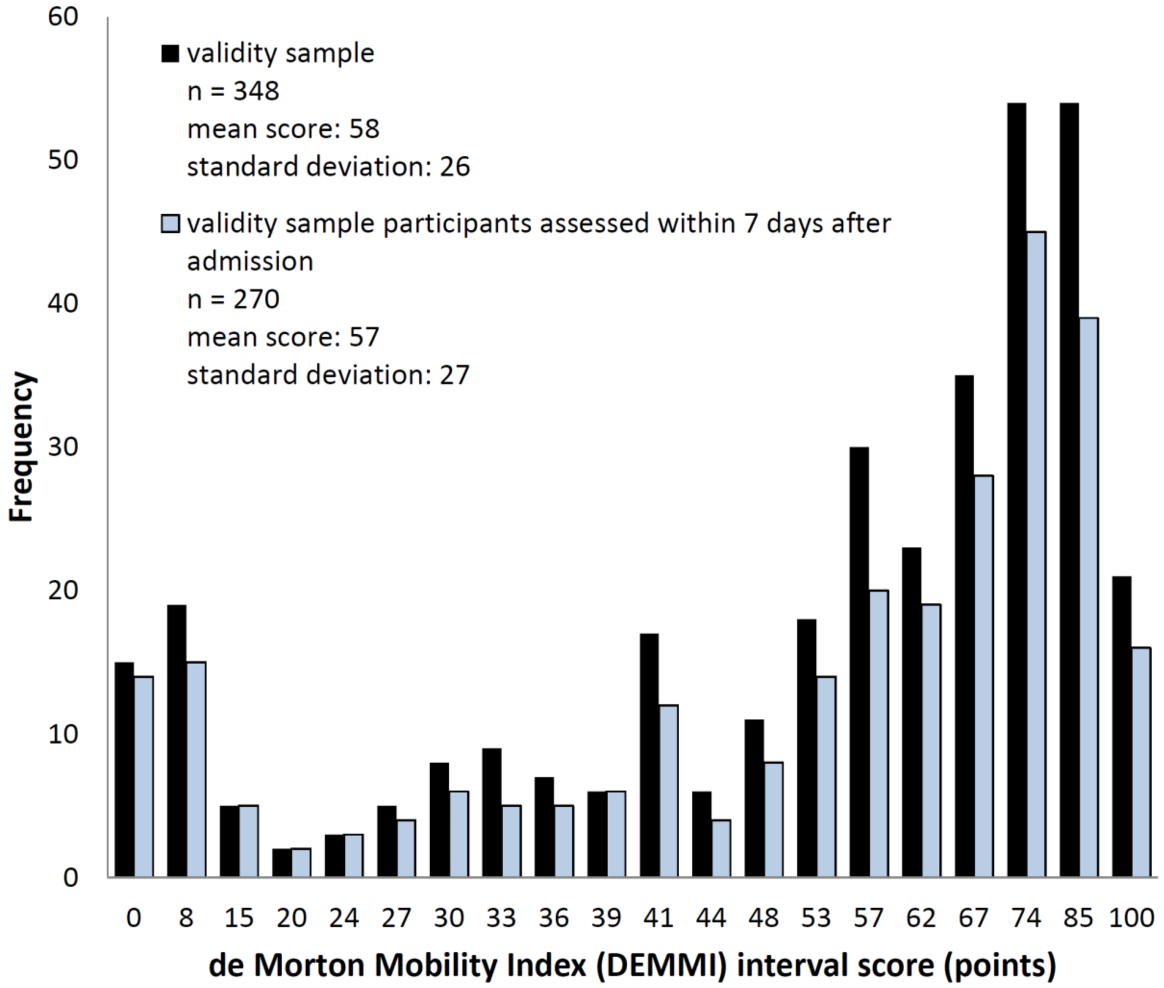

Additional file 2: Additional results

Item logit location

Item logit location (with 95% confidence intervals) and item hierarchy of difficulty for the sample of rehabilitation inpatients with neurological conditions and the original older acute geriatric sample DEMMI data (de Morton et al. 2008).

A high positive logit location (e.g., standing on toes) indicates harder item difficulty compared to a negative logit location (e.g., sit unsupported).

Deviation from the original hierarchy is indicated by non-overlapping 95% confidence bands.

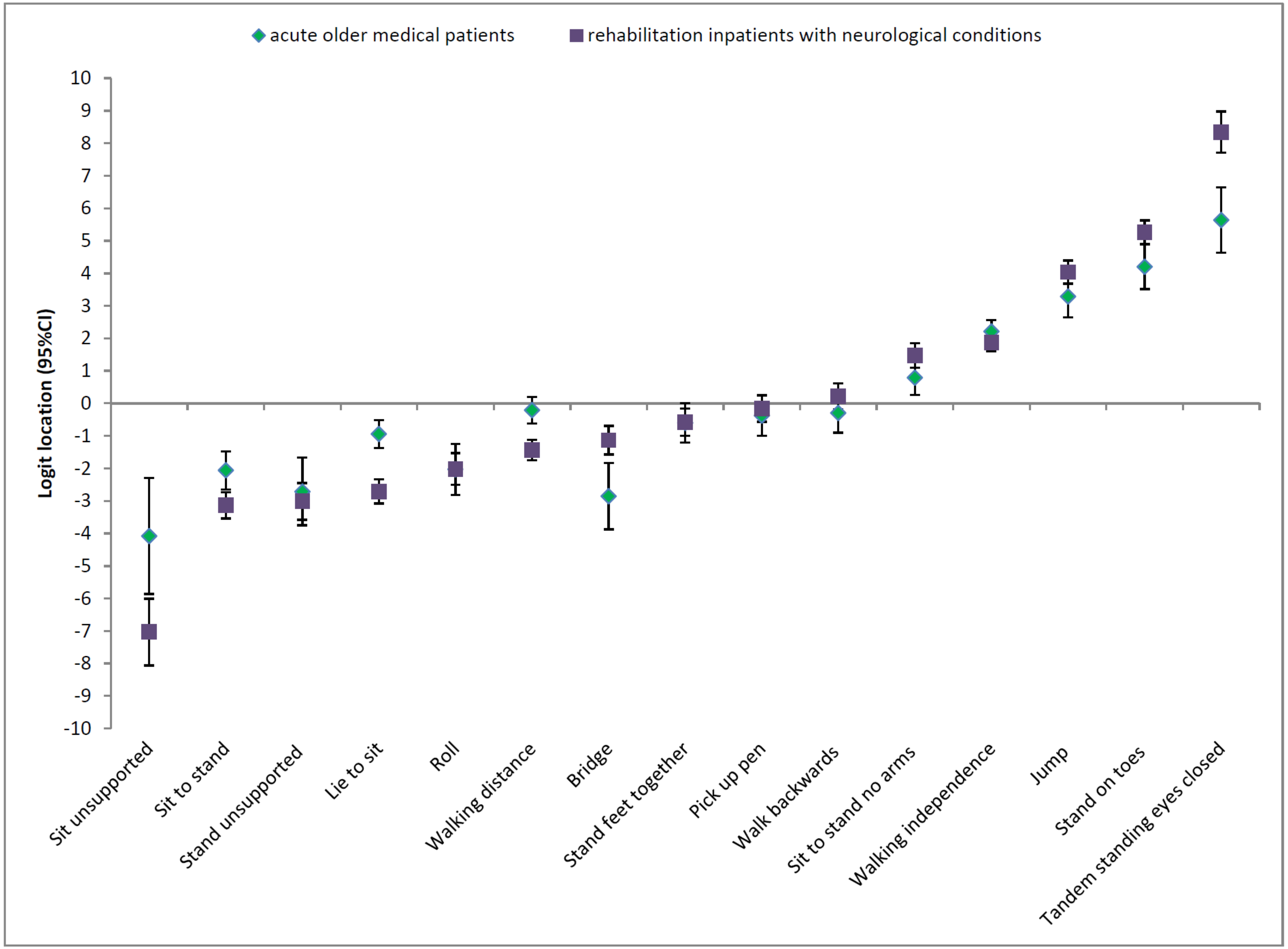

Additional file 2: Additional results

Person-item distribution of the de Morton Mobility Index

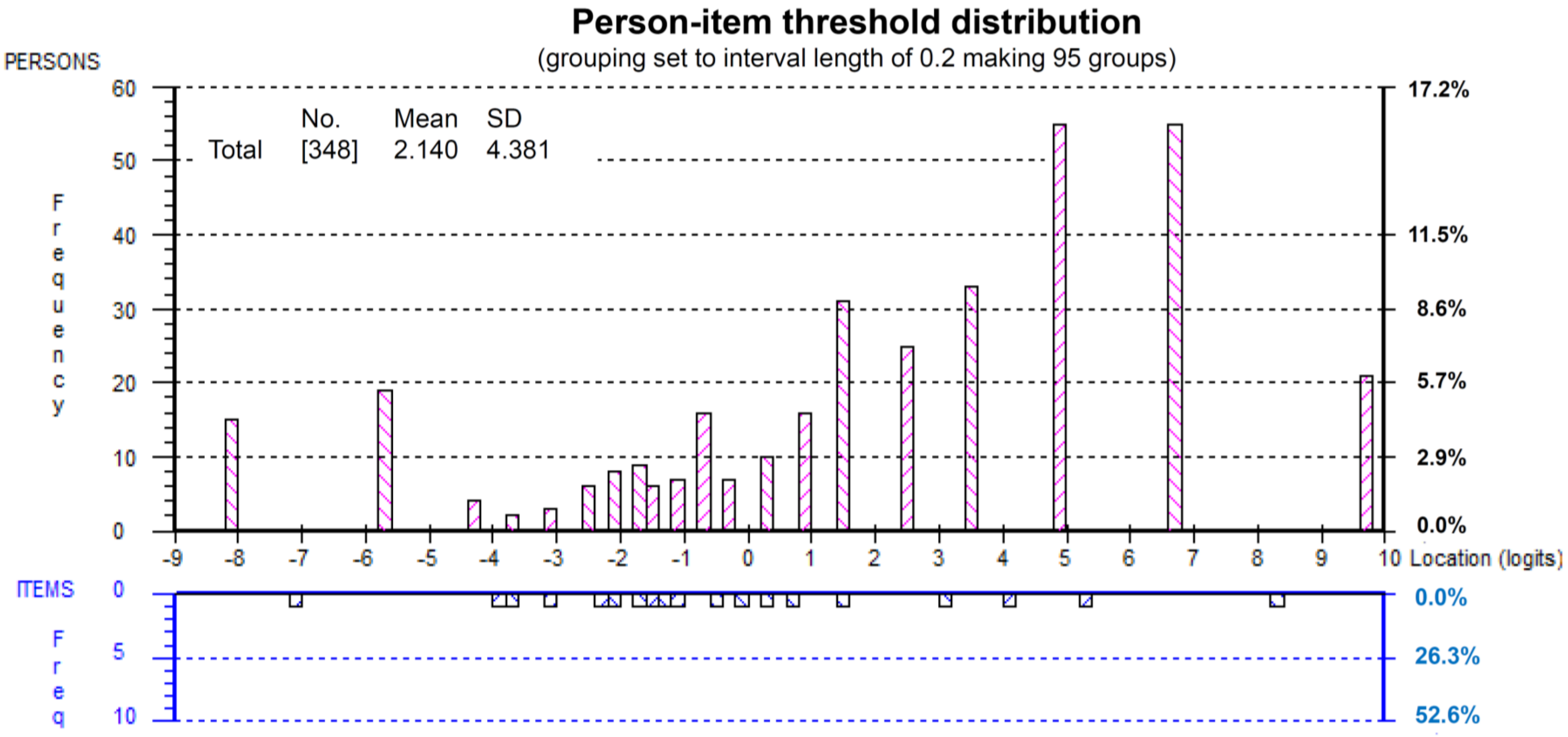

Additional file 2: Additional results

Item characteristic curves (ICC) and Differential Item Functioning (DIF) for item #2 'roll to one side' (uniform)

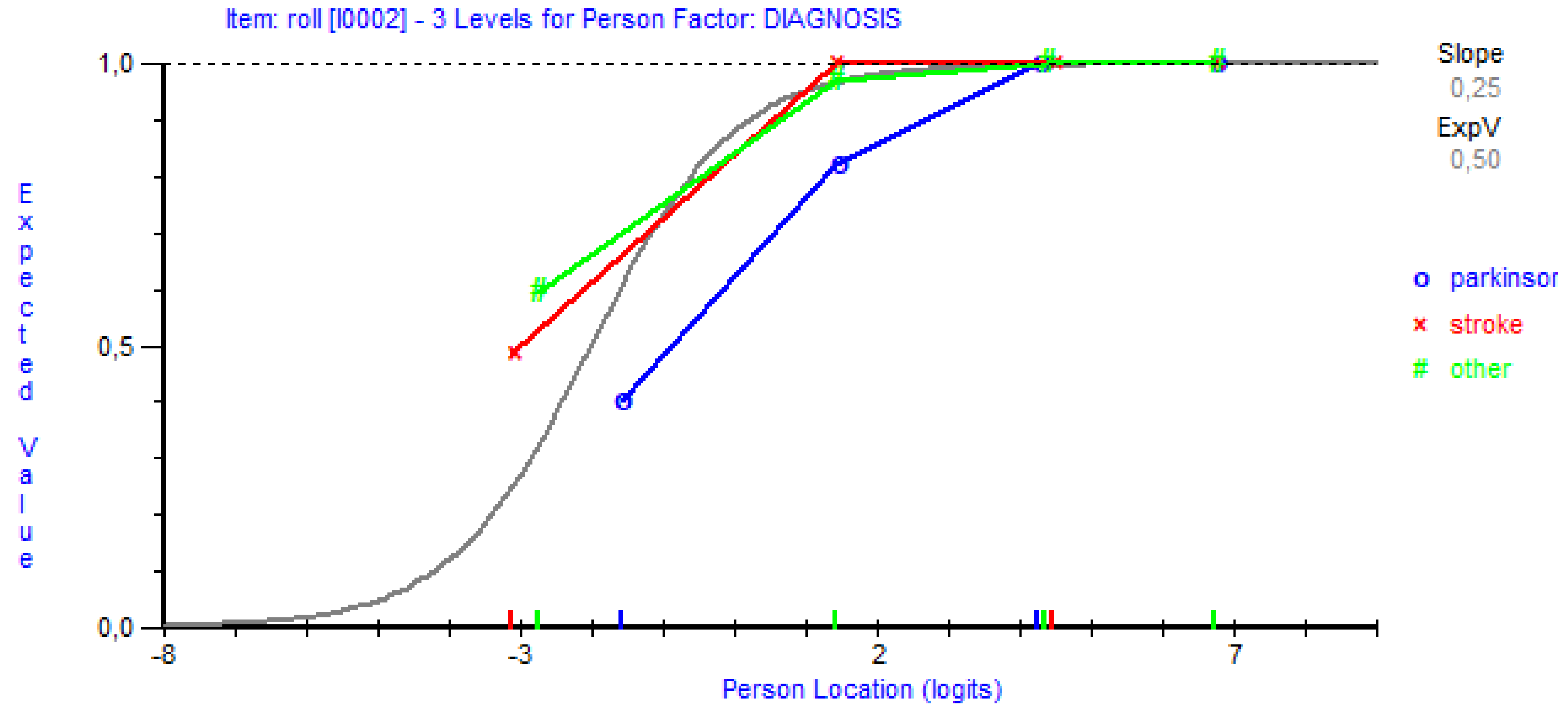

Additional file 2: Additional results

Item characteristic curves (ICC) and Differential Item Functioning (DIF) for item #15 'jump' (uniform)

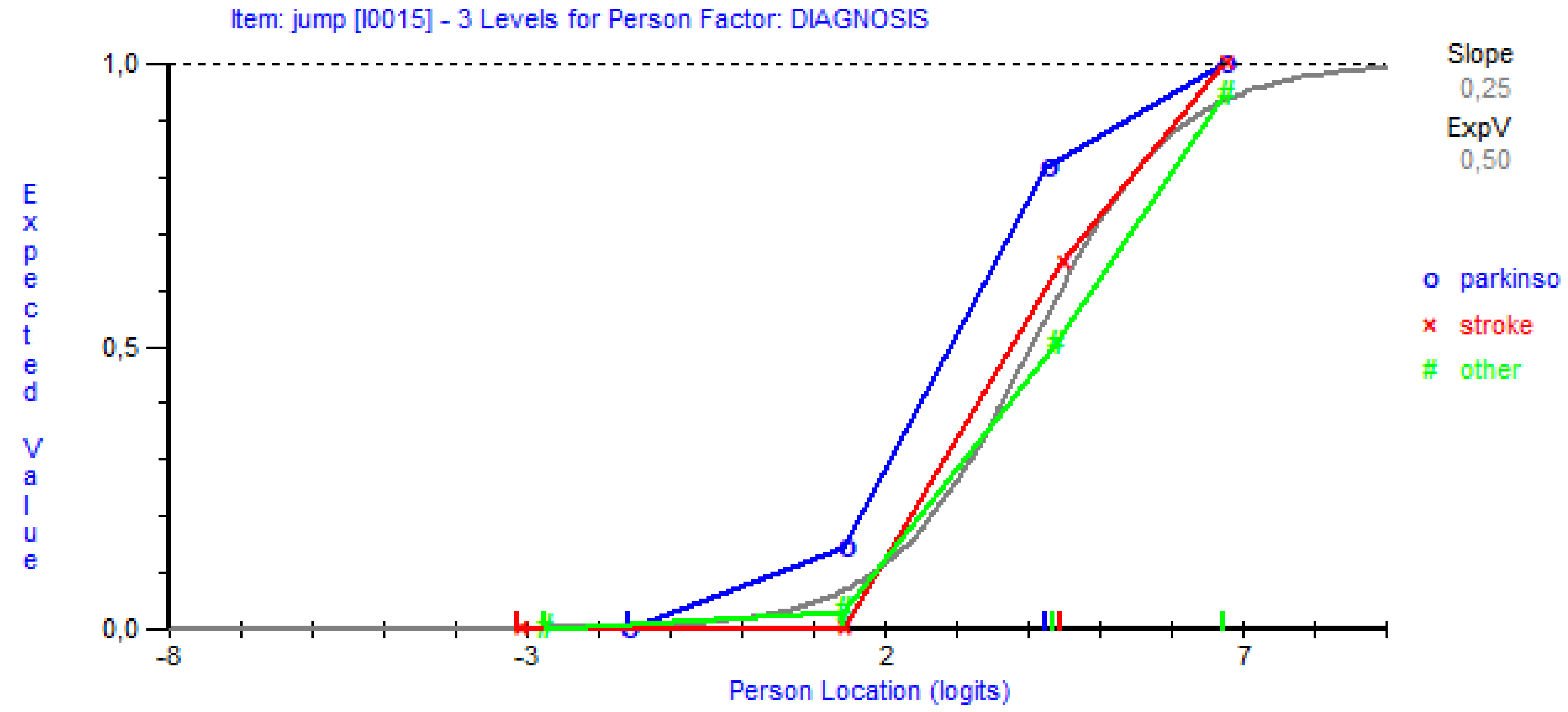

Additional file 2: Additional results

Results on known-groups validity of the de Morton Mobility Index

No.	Hypotheses	Observed mean DEMMI scores (points) according to clinical groups		Statistical significance (Mann-Whitney U test, 1-fold)	Hypothesis confirmed
		Clinical groups	DEMMI mean ± SD (range) score		
9	In the sample of ambulatory (FAC ≥ 3; n = 280) participants: A statistically significant mean difference between participants walking without a walking aid and participants walking with a walking aid.	No walking aid (n = 161)	78 ± 13 (24 – 100)	U = 1127; P < 0.01	Yes
		With walking aid (n = 119)	53 ± 11 (15 – 74)		
10	A statistically significant mean difference between independently ambulatory (FAC ≥ 4) and dependently ambulatory/non-ambulatory (FAC ≤ 3) participants.	Independent (n = 230)	72 ± 15 (33 – 100)	U = 838; P < 0.01	Yes
		Dependent/non-ambulatory (n = 118)	29 ± 19 (0 – 67)		
11	In the sample of participants with FIM data available (n = 325): A statistically significant mean difference between participants who can climb stairs independently (FIM stair item 6 – 7 points) and those participants who are dependent or cannot climb stairs (FIM stair item 1 – 5 points).	Independent (n = 66)	80 ± 11 (53 – 100)	U = 2408; P < 0.01	Yes
		Dependent/not able to climb stairs (n = 259)	51 ± 26 (0 – 100)		

SD: standard deviation; DEMMI = de Morton Mobility Index; FIM = Functional Independence Measure; FAC = Functional Ambulation Categories

Additional file 2: Additional results

Agreement between the two raters per DEMMI item

No.	Item	Agreement (%) with 95% CI	Kappa (ƙ) with 95% CI
1	Bridge	92 (85 – 96)	0.70 (0.53 – 0.86)
2	Roll onto side	94 (88 – 97)	0.74 (0.57 – 0.91)
3	Lying to sitting	88 (81 – 93)	0.75 (0.63 – 0.88)
4	Sit unsupported in chair	99 (94 – 100)	0.85 (0.64 – 1.00)
5	Sit to stand from chair	96 (91 – 99)	0.92 (0.85 – 0.99)
6	Sit to stand without using arms	88 (81 – 93)	0.72 (0.60 – 0.85)
7	Stand unsupported	95 (90 – 98)	0.82 (0.67 – 0.96)
8	Stand feet together	84 (77 – 90)	0.62 (0.48 – 0.76)
9	Stand on toes	80 (72 – 87)	0.49 (0.33 – 0.66)
10	Tandem stand with eyes closed	86 (79 – 92)	0.29 (0.05 – 0.53)
11	Walking distance	92 (85 – 96)	0.82 (0.71 – 0.93)
12	Walking independence	89 (83 – 94)	0.88 (0.82 – 0.94)
13	Pick up pen from floor	92 (86 – 96)	0.81 (0.70 – 0.92)
14	Walk 4 steps backwards	88 (81 – 93)	0.72 (0.59 – 0.84)
15	Jump	87 (80 – 92)	0.74 (0.62 – 0.85)

Additional file 2: Additional results

Administration time of the DEMMI according to scale range

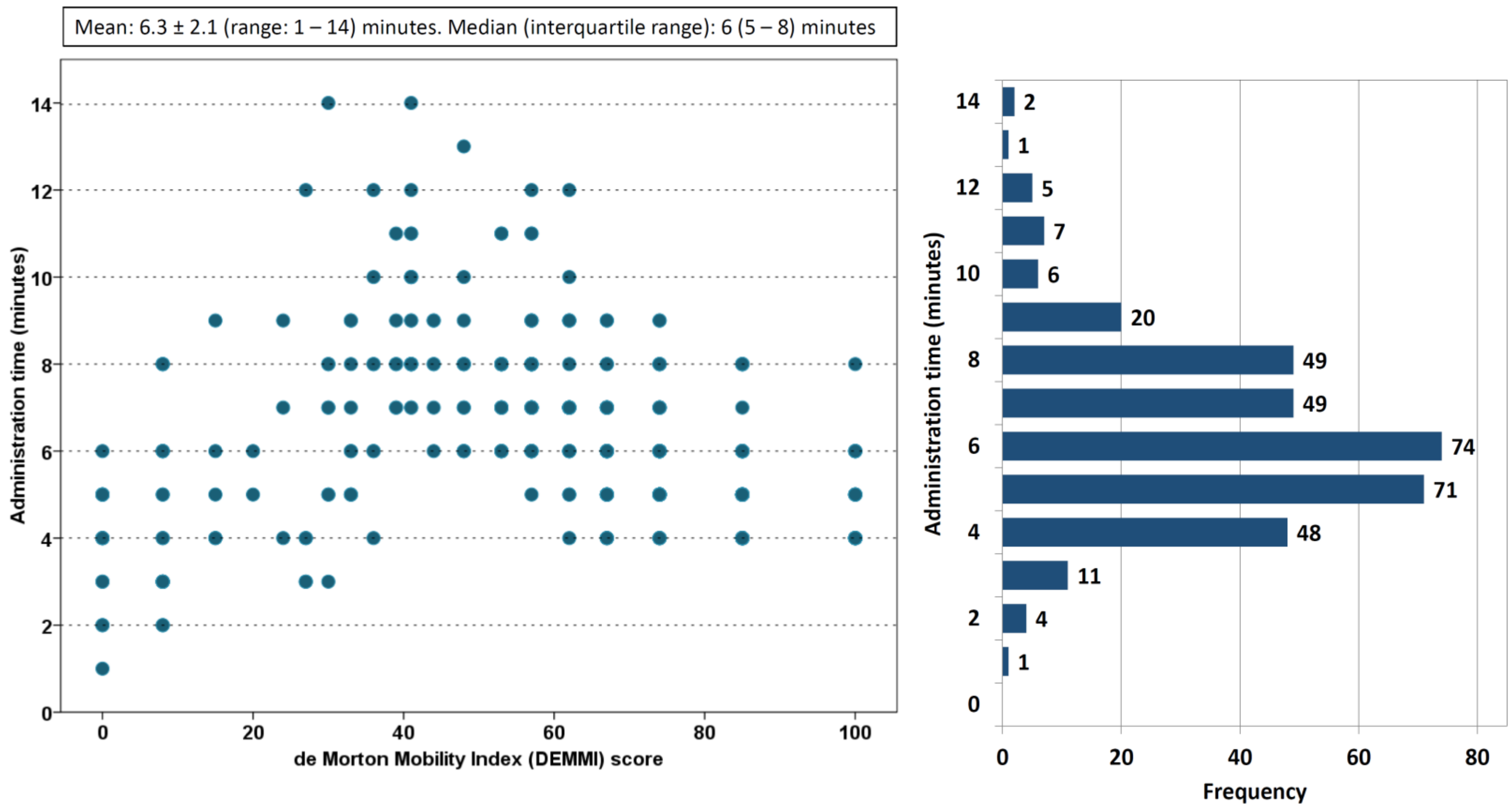

Supplement: Supplementary file 2 — Additional file 2. Additional results. [file 12883_2021_2327_MOESM2_ESM.pdf]
